# Combining computational neuroscience and body sensor networks to investigate Alzheimer’s disease

**DOI:** 10.1186/1471-2202-13-S1-P178

**Published:** 2012-07-16

**Authors:** Jeroen Bergmann, Newton Howard

**Affiliations:** 1 Medical Engineering Solutions in Osteoarthritis Centre of Excellence, Imperial College, London, W68RF, UK; 2 Massachusetts Institute of Technology, Cambridge, MA, 02139, USA

## 

Alzheimer’s disease is a syndrome of acquired cognitive defects that interferes with normal brain function. While its biochemical effects are well documented, the cause of Alzheimer’s disease is still not known and the best available interventions remain merely symptomatic. Different treatment modalities have been explored over the last few decades. Pharmaceutical interventions currently consist of medicines that focus on the neuropsychiatric symptoms and disease-modifying treatments. The importance of early intervention to the efficacy of treatments has led to an emphasis on detection and diagnosis in clinical research, and techniques using imaging, biomarkers and genetic information as tools for early detection have become prevalent. However, multifaceted non-invasive screening tools that incorporate computational algorithms and do not rely on imaging are not being widely developed. This paper argues that a computation method originally developed to explain mental processes can be adapted to assist in the early detection of Alzheimer’s disease. Temporal changes in behaviour and speech are occurring in the early stages of the disease. A Body Sensor Network (BSN) can be utilized to collect temporal information during everyday living which can be further processed with an algorithm that allows for natural randomness. This method shows that novel non-invasive screening tools for Alzheimer’s disease can be devised based on measuring real-life behaviour.

## Conclusions

It has been shown that BSNs can be utilized to measure a range of activities. A MSI classification algorithm can subsequently categorize the temporal changes during ADL activities, while allowing for natural occurring randomness [1]. The BSN can be unobtrusive to allow for monitoring over longer periods of time [2]. The technique outlined here can be used for diagnostic purposes, but it can also be implemented to screen for treatment effects of new and existing interventions.

## References

[B1] Howard N Guidere M Computational Methods for Clinical Applications: An Introduction Functional Neurology, Rehabilitation, and Ergonomics 1998 1 NewYork: Springer-Verlag 237 250 20112. Bower JM, Beeman D: The Book of Genesis, 2nd Edition

[B2] Bergmann JHM McGregor AH Sensor design: what do patients want? The 2nd International Conference on Ambulatory Monitoring of Physical Activity and Movement 2011 Glasgow

